# Effects of School-Based Physical Activity Programs on Health-Related Physical Fitness of Korean Adolescents: A Preliminary Study

**DOI:** 10.3390/ijerph18062976

**Published:** 2021-03-14

**Authors:** Eui-Jae Lee, Wi-Young So, Hyun-Su Youn, Jooyoung Kim

**Affiliations:** 1 Department of Physical Education, Sogang University, Seoul 04107, Korea; freedom_jae@hanmail.net; 2 Sports Medicine Major, College of Humanities and Arts, Korea National University of Transportation, Chungju-si 27469, Korea; wowso@ut.ac.kr; 3 Department of Physical Education, College of Education, WonKwang University, Iksan-si 54538, Korea; 4 Office of Academic Affairs, Konkuk University, Chungju-si 27478, Korea

**Keywords:** adolescents, body mass index, physical activity, physical fitness, school-based program

## Abstract

As adolescents spend the majority of their time focused on exams and assignments, they do not have sufficient time to engage in physical activity; this lack of physical activity is an important public health concern. This study aimed to investigate how school-based physical activity programs affect the health-related physical fitness of adolescents in the Republic of Korea. For this study, a total of 120 high school students participated in a school-based physical activity program that included badminton and table tennis for 15 weeks each (35 min/day, three times a week), with a total of 30 weeks for one academic year. The parameters for health-related physical fitness measured muscle strength (handgrip strength), power (standing long jump), cardiorespiratory fitness (shuttle run test), flexibility (sit and reach), body mass index (BMI), and the total score. The results revealed a statistically significant improvement in muscle strength (*p* < 0.001), power (*p* < 0.001), cardiorespiratory fitness (*p* < 0.001), flexibility (*p* = 0.005), and the overall health-related physical fitness score (*p* = 0.001). However, students’ BMI showed no significant difference before and after participation (*p* = 0.825). The results of this study indicated that school-based physical activity programs can have a positive effect on the health-related physical fitness of adolescents.

## 1. Introduction

The lack of physical activity in adolescents is a commonly experienced problem worldwide and, thus, is an important public health concern [1,2]. As adolescents spend the majority of their time focused on exams and assignments, they do not have enough time to participate in physical activities. In addition, sedentary lifestyles have become the norm among adolescents, further reducing the level of physical activity; reports claim that this lifestyle is believed to contribute to the continuous increase in the percentage of overweight and obese adolescents [3,4]. This, along with cardiovascular and metabolic risk factors, increases musculoskeletal pain, injuries, fractures, and sleep apnea, giving rise to low self-esteem, poor quality of life, and health-related economic costs among adolescents [5,6].

Therefore, to promote public health and adolescent health, the importance of physical activity should be emphasized, and programs that can help improve their skill and physical fitness must be applied. The recommended minimum level of physical activity for adolescents is moderate to vigorous activity for at least 20 min per session, three times a week [7]. Several studies have proposed school-based physical activity programs to encourage physical activity in adolescents, as schools are an ideal environment for implementing physical activity programs, and these programs are known to be effective in promoting physical fitness [8,9,10]. In addition, adolescents are most active during school days, and schools provide access to physical activities in a manner that is independent of academic background or socioeconomic status [8,11]. Furthermore, school-based physical activities ensure the exposure of a maximum number of adolescents to the intervention, and it is relatively easy to increase the number of adolescents participating in these activities. In this way, overweight and obese adolescents can also participate in these activities without a sense of disliking such activities [7]. According to a previous study, school-based health education programs are associated with the enhancement of self-concept and self-worth in disadvantaged populations, indicating that these programs can be better implemented in school-based settings rather than in other environments [12]. Several studies have reported that a school-based health program that promotes an active lifestyle in adolescents can contribute to improving the health-related quality of life by reducing the time spent watching television as well as by improving physical fitness parameters, such as muscle strength and cardiovascular fitness [7,13,14].

Several studies have also reported that school-based intervention can help improve physical fitness of adolescents [15,16,17]. Ardoy et al. [15] reported that school-based physical activity of adolescents significantly improved their flexibility and speed–agility, including aerobic fitness. Yoshimoto et al. [16] emphasized that the body fat of adolescent girls was significantly reduced when school-based exercises, including squats, were regularly performed as part of their after-school activities. In addition, school-based exercise was recently found to have a positive effect on the changes in body composition and muscular and physical aerobic performance of overweight adolescent girls [17]. However, such investigation has not been conducted in the Republic of Korea thus far. Therefore, this study aimed to investigate how school-based physical activity programs, including badminton and table tennis, affect the health-related physical fitness of adolescents in the Republic of Korea.

## 2. Methods

### 2.1. Participants and Design

Based on a paired *t*-test design and an anticipated statistical power (1-β) of 0.80, in addition to an alpha error probability of 0.05 with an effect size of 0.40 as per Cohen’s table [18], the required sample size was determined to be 52 participants (G-power program 3.1.3, Heinrich-Heine-University, Düsseldorf, Germany). In this study, we used a sample size comprising 155 participants in order to factor in those who would be excluded from the analysis. Participants included 155 high school students who applied for a school-based physical activity program in Goyang-si province, Republic of Korea. Participants included only those students who did not participate in any regular exercise program (other than physical activity in school or after-school classes) in the past three months, did not have any disease at the time (including musculoskeletal injury), and did not use any drugs that could influence the results of the study. The students who did not meet these inclusion criteria were excluded from the study.

Prior to the start of the study, participants signed a written informed consent form. Before they participated in any physical activity programs (including badminton or table tennis) each semester, the baseline of the physical fitness of participants was measured. Physical fitness was measured again after the end of the program. Among the 155 applicants, only 120 high school students (65 males and 55 females; age: 18.95 ± 0.23 years; height: 167.69 ± 7.76 cm; weight: 59.2 ± 12.54 kg) who participated in all of the three-times-a-week sessions for one year were selected for the final analysis. The remaining 35 students were excluded for the following reasons: low attendance in the sessions and withdrawal due to injury. This study was approved by WonKwang University’s institutional review board (WKIRB-202012-HR-081).

### 2.2. School-Based Physical Activity Program

During the study period, the program was conducted in the morning from 8:00 am to 8:35 pm, with badminton and table tennis in the first and second semesters, respectively, from March 2018 to March 2019. The choice of the physical activities was made based on the survey of students’ preferences. Before and after 35 min of exercise, warm-up and cool down were performed for 10 min each, including light jogging and stretching. Each type of physical activity was performed for 15 weeks (35 min/day, three times a week), and the entire program ran for 30 weeks for one academic year. During the first 3 weeks, repeated practice of basic skills was conducted, and then, badminton or table tennis games were held.

### 2.3. Health-Related Physical Fitness Test

The parameters of the health-related physical fitness test were based on the physical activities promotion system (PAPS) conducted in Korean high schools. The PAPS includes muscle strength, power, cardiorespiratory fitness, flexibility, BMI, and a total score that sums these five parameters. Muscle strength was measured using handgrip strength, wherein participants had their feet attached to the floor and spread appropriately for an upright posture. After holding the dynamometer (TKK-5401, TAKEI, Niigata, Japan), with appropriate adjustments, measurements were taken for the left and right hand—twice—and the mean value was used as the final value [19,20]. Power was measured through a standing long jump, where participants jumped forward from a starting line as far as possible. The researcher measured the straight-line distance from the nearest point of contact by any part of the participant’s body to the floor. Measurements were taken twice, and the best measurement record was used [19,20]. Cardiorespiratory fitness was measured using a shuttle run test. Measurements were taken in a gym with a flat, non-slippery floor, where the researchers placed asphalt cones at both ends to mark the starting and finishing points, measuring a 20 m distance. After a soundcheck and warm-up exercise, participants waited for the signal at the starting point and then started running on the beeper indicating “start.” Participants ran to the opposite side before the next beep sound and waited for the next beep before running back to the other side. This process was repeated as many times as possible within the participant’s capacity. If participants failed to run to the beep, they received one warning, and after two warnings, the test was ended [19,20]. Flexibility was measured by the sit and reach test, wherein participants took off their shoes and sat down with their feet touching the measuring instrument. Both palms were straightened, and the left palm was placed on the back of the right hand so that they overlapped. On the instruction to start, the chest was pushed forward and the upper body was bent, with hands reaching below the scale of the measuring instrument. When the upper body was bent forward, the researcher gently pressed the knee to ensure that the knee was not bent. Measurements were recorded while maintaining the bent posture for more than 3 s. This was performed for two runs, and the mean value was recorded. Measurements were retaken when the knee was bent or rebound was used [19,20]. BMI was calculated by dividing the body weight (kg) by the height squared in meters (kg/m^2^). Wearing thin shirts and shorts and their socks removed, participants placed two feet on the plate of a stadiometer (GL-150R; G-Tech International, Co., Ltd., Uijeongbu, Korea), and the body weight were measured. Participants’ measurements were taken after they had fasted for eight hours to obtain the accurate body weight. They were asked to refrain from performing vigorous exercise and physical activities or taking a long bath the day before the measurement [19,20]. Finally, the total health-related physical fitness score was calculated by summing the scores obtained for muscle strength, power, cardiorespiratory fitness, flexibility, and BMI.

### 2.4. Statistical Analysis

For the statistical analysis, SPSS software (version 18.0, IBM Corp., Armonk, NY, USA) was used. All data were presented as mean and standard deviation. A paired *t*-test was performed to compare changes in health-related physical fitness pre-and post-intervention. The statistical significance level was set at 0.05.

## 3. Results

Table 1 presents the participant characteristics by sex, and Table 2 presents the changes in physical fitness variables. In terms of muscle strength (hand grip strength), the pre-program mean value was 33.39 ± 11.05 kg, and the post-program mean value was 42.34 ± 11.52 kg, indicating an improvement of more than 9 kg and a statistically significant difference (*p* < 0.001). In terms of power (standing long jump), the pre-program mean value was 168.90 ± 38.36 cm, while the post-program mean value was 178.24 ± 41.62 cm, indicating an improvement of about 10 cm and a statistically significant difference (*p* < 0.001). In terms of cardiorespiratory fitness (shuttle run test), the pre-program mean value was 42.71 ± 22.90 reps, while the post-program mean value was 54.51 ± 18.56 reps, indicating an improvement of about 12 reps, with a statistically significant difference (*p* < 0.001). In terms of flexibility (sit and reach), the pre-program mean value was 13.07 ± 8.91 cm, and the post-program mean value was 14.73 ± 8.15 cm, indicating an improvement by about 1.7 cm or more, showing a statistically significant difference (*p* = 0.005). For the BMI, the pre-program mean value was 21.04 ± 3.55 kg/m^2^, and the post-program mean value was 21.08 ± 3.68 kg/m^2^, showing no statistically significant difference (*p* = 0.825). For the total health-related physical fitness score, the pre-program mean value was 53.09 ± 14.05, and the post-program mean value was 56.29 ± 12.71, indicating an improvement by about three points or more, showing a statistically significant difference (*p* = 0.001).

## 4. Discussion

This study aimed to investigate how school-based physical activity programs affect the health-related physical fitness of adolescents in the Republic of Korea. To the best of our knowledge, this is the first of such a study on Korean adolescents. Our study confirmed that there were statistically significant improvements in health-related physical fitness parameters after participation in a school-based physical activity program.

Recent studies have actively proposed the implementation of school-based physical activity programs to increase the opportunities for adolescents to participate in physical activities [21,22,23]. Kelly et al. [21] reported that health promotion interventions in schools can increase students’ access to physical activity opportunities. Demetriou et al. [22] reported that in order to increase and maintain the level of physical activity in adolescents, motivation for the same goal must be sustained, and promoting physical activities in school settings is effective in enhancing this motivation. Silva et al. [23] discussed that, with regard to physical activities, school-based programs have the potential to improve student health and academic performance, while creating opportunities for adolescents to engage in school activities. Several studies have reported that such programs are effective in increasing the time devoted to physical activities by adolescents and in improving physical health [24,25]. For example, Isensee et al. [24] showed that German adolescents who received school-based intervention showed an increase in the time devoted to participation in physical activities compared to students who did not undergo such intervention. Andrade et al. [25] reported that school-based health promotion interventions could improve the physical health of Ecuadorian adolescents and minimize the decrease in their level of physical activity.

Some other previous studies [26,27,28] supported the findings observed in this study, reporting that school-based intervention had a positive effect on changes in health-related physical fitness. Kelishadi et al. [26] showed that after 6 weeks of school-based physical activities, including badminton, soccer, and volleyball, the maximum oxygen intake of Iranian adolescents increased compared to pre-participation. Giannaki et al. [27] showed that Cypriot adolescents who underwent school-based circuit training for 8 weeks showed a significant improvement in cardiorespiratory fitness and a significant decrease in total body fat. In addition, Martin-Smith et al. [28] reported that a school-based sprint interval training program for 4 weeks significantly increased cardiorespiratory fitness and physical activities of Scottish adolescents. Ardoy et al. [15] reported that school-based physical activities of Spanish adolescents, undertaken for 16 weeks, significantly changed their physical fitness and were sufficiently stimulating to particularly improve aerobic fitness; an indicator of cardiovascular health.

In this study, the school-based physical activity program included badminton and table tennis. Badminton involves jumping, changing directions, quick arm movement with explosive movements over a short time while holding a racket [29]. Similarly, table tennis requires holding the racket and handling the ball while stroking in a short time [30]. Furthermore, both badminton and table tennis require aerobic capacity, which helps maintain a variety of skills required during the game [31,32]. The characteristics of the movement and physiological changes required in badminton and table tennis contribute to the improvement of adolescents’ performance in handgrip strength, standing long jump, shuttle run test, and sit and reach test.

In this study, differently from the other factors measured, there was no statistically significant difference in BMI before and after participation in the program. Similarly, Dobbins et al. [7], Guerra et al. [33], and Kelishadi et al. [26] reported that school-based physical activity intervention had no significant effect on BMI. In addition, Harris et al. [34] reported, through a meta-analysis, that school-based physical activity intervention caused positive changes in other health factors; however, BMI results did not show improvements.

Although in the study by Kelishadi et al. [26] there was no significant change in the BMI after school-based physical activities, the waist–hip ratio (WHR) and body fat percentage were significantly decreased compared to pre-activities, thus, showing changes in body composition. One limitation of this study is the fact that only BMI was measured to interpret the results. BMI is generally used for the evaluation of obesity; however, it is limited in its ability to reflect body composition and body image information in detail [35,36]. Therefore, in this study, clearly determining how the body composition information of adolescents changed after their participation in the program is difficult when using only BMI information. Nevertheless, it is worth noting that no significant changes were observed in BMI because most scores fell in the normal range and were maintained throughout the program. Hence, it is possible that participation in the program helped maintain the BMI. In future studies, more indices, such as the WHR and body fat percentage, should be included to investigate the changes in body composition.

This study has other limitations. First, there was no control group. Only the differences between the pre- and post-participation were analyzed. In future studies, well-designed randomized controlled trials are recommended. Second, this study was conducted for a single high school, and, thus, the sample size was small, making it difficult to generalize the results of this study. In future research, it is necessary to increase the sample size. Third, although it is important to present the impact of sex, age, and maturation on intervention effects, we did not investigate these aspects in this study. Finally, other factors known to affect the level of physical activity, such as sex, regular physical education classes, parental support, nutritional intake, and psychological status of the adolescents participating in this study, were not analyzed [37].

## 5. Conclusions

In summary, the results of this study confirmed that school-based physical activity programs can have a positive impact on the health-related physical fitness of adolescents. These results are expected to assist health and education professionals to plan or make decisions on strategies that can promote physical activities in schools. In future research, the effects of school-based physical activity programs for adolescents should be considered not only for physical outcomes but also for learning performance, mental health, lifestyle, and behavior.

## Figures and Tables

**Table 1:** Participant characteristics by sex.

Variables	Boys (*n* = 65)	Girls (*n* = 55)	*t*	*p*
Age (years)	18.94 ± 0.24	18.98 ± 0.23	−1.005	0.317
Height (cm)	172.68 ± 6.42	161.890 ± 4.47	10.468	<0.001 ***
Weight (kg)	65.91 ± 6.42	51.56 ± 6.49	7.555	<0.001 ***
Hand grip strength (kg)	40.39 ± 9.96	25.25 ± 5.05	10..200	<0.001 ***
Standing long jump (cm)	193.34 ± 29.248	140.45 ± 26.163	10.322	<0.001 ***
Shuttle run test (repetitions)	53.22 ± 22.67	30.47 ± 16.221	−26.199	<0.001 ***
Sit and reach (cm)	11.70 ± 8.18	14.65 ± 9.52	−2.951	0.072
Body mass index (kg/m^2^)	22.18 ± 4.05	19.72 ± 2.254	4.009	<0.001 ***
Health-related physical fitness total score (points)	51.720 ± 14.492	54.69 ± 13.473	−2.972	0.252

*** *p* < 0.001; tested by paired *t*-test.

**Table 2:** Changes in physical fitness variables after school-based physical activity program.

Variables	Pre-Program	Post-Program	*t*	*p*
Hand grip strength (kg)	33.39 ± 11.05	42.34 ± 11.52	−16.561	<0.001 ***
Standing long jump (cm)	168.90 ± 38.36	178.24 ± 41.62	−4.588	<0.001 ***
Shuttle run test (reps)	42.71 ± 22.90	54.51 ± 18.56	−6.966	<0.001 ***
Sit and reach (cm)	13.07 ± 8.91	14.73 ± 8.15	−2.837	0.005 **
Body mass index (kg/m^2^)	21.04 ± 3.55	21.08 ± 3.68	−0.221	0.825
Health-related physical fitness total score (points)	53.09 ± 14.05	56.29 ± 12.71	−3.398	0.001 **

** *p* < 0.01, *** *p* < 0.001; tested by paired *t*-test.

## Data Availability

The data presented in this study are available on request to the authors. Some variables are restricted to preserve the anonymity of study participants.
